# Quality Improvement in Critical Care: Selection and Development of Quality Indicators

**DOI:** 10.1155/2016/2516765

**Published:** 2016-07-14

**Authors:** Carla A. Chrusch, Claudio M. Martin, The Quality Improvement in Critical Care Project

**Affiliations:** ^1^Department of Critical Care Medicine, University of Calgary, Calgary, AB, Canada T2V 1P9; ^2^Department of Medicine, University of Western Ontario, London, ON, Canada N6A 4G5; ^3^The Quality Improvement in Critical Care Project, Canada

## Abstract

*Background*. Caring for critically ill patients is complex and resource intensive. An approach to monitor and compare the function of different intensive care units (ICUs) is needed to optimize outcomes for patients and the health system as a whole.* Objective*. To develop and implement quality indicators for comparing ICU characteristics and performance within and between ICUs and regions over time.* Methods*. Canadian jurisdictions with established ICU clinical databases were invited to participate in an iterative series of face-to-face meetings, teleconferences, and web conferences. Eighteen adult intensive care units across 14 hospitals and 5 provinces participated in the process.* Results*. Six domains of ICU function were identified: safe, timely, efficient, effective, patient/family satisfaction, and staff work life. Detailed operational definitions were developed for 22 quality indicators. The feasibility was demonstrated with the collection of 3.5 years of data. Statistical process control charts and graphs of composite measures were used for data display and comparisons. Medical and nursing leaders as well as administrators found the system to be an improvement over prior methods.* Conclusions*. Our process resulted in the selection and development of 22 indicators representing 6 domains of ICU function. We have demonstrated the feasibility of such a reporting system. This type of reporting system will demonstrate variation between units and jurisdictions to help identify and prioritize improvement efforts.

## 1. Introduction

Critically ill patients experience a high burden of disease and providing care for these patients is expensive and complex. The stresses placed on critical care services continue to escalate from increases in both the numbers of patients and expectations regarding the provision of safe, quality care. There is concern that demand will outstrip our already overburdened human resources [1]. These demands are further compounded by the perpetual challenge of achieving high quality care without excessive resource use. Approaches to measuring and increasing the application of best practice in individual disease processes, such as sepsis, have been developed and to some extent also measure the function of a system [2]. However, a more global description and balanced assessment of critical care performance is needed in order to understand how to make it better.

Part of the recommendations in To Err Is Human: Building a Safer Health System [3] was the setting of performance standards and consistency across organizations. This was reiterated at the PrOMIS (Prioritizing the Organization and Management of Intensive care Services in the United States) Conference that identified the lack of a standardized, national performance measurement of critical care services as a major problem [4]. A major challenge surrounding the development of such measures for critical care was the lack of standardized definitions that included specification of the population at risk and the period of exposure to risk, such as device days, for example. Another challenge to be addressed is the development of methodologies for dealing with uncommon or rare events [5]. Pediatric [6, 7] and adult critical care [8, 9] have begun the process of developing quality indicators.

Sound management decisions need to be based on an understanding of past performance, current need and utilization, and anticipated population needs. A national ICU database with consistent definitions and nomenclature would provide a mechanism for comparing characteristics and performance between units and regions over time. The objective of this project was to select and refine indicators for a critical care scorecard encompassing multiple domains of intensive care unit function as well as a mechanism for comparing the characteristics and performance between and within units, health care systems, and regions over time.

## 2. Methods

### 2.1. Quality Indicator Selection and Development

Canadian healthcare organizations with established critical care datasets were invited to participate. A conference was held on February 9–12, 2005, with plenary sessions to provide an overview of current report card type projects and potential domains. Participants at the inaugural conference represented 7 healthcare organizations across 5 provinces and included 9 intensivists, most of whom held senior administrative posts, 5 administrators (2 of whom were also nurses), 2 information technology experts, and a quality improvement consultant. Participants had considerable expertise across all facets of critical care including clinical care, quality improvement, epidemiology, and administration. Discussions were facilitated by the quality improvement consultant.

The group reached consensus on the domains and principals for indicator selection. The six domains of a critical care unit care and function selected were safe, timely, effective, efficient, patient/family satisfaction, and staff work life. The guiding principles for the selection of quality indicators agreed upon were the following:

- The intended audience is providers of critical care services.
- The perspective is for management and quality improvement.
- Indicators will
  - be chosen based on usefulness, feasibility, and reliability,
  - be action enabling,
  - represent a mixture of processes, outcomes, and cost,
  - reflect present performance with a mix of lead and lag indicators,
  - be based on available evidence, or in the absence of high-level evidence on benchmarks; when using a benchmark, the target will be set at a high level as opposed to an average or median target.
- As much as possible, previously validated indicators and definitions will be used.

Ultimately, five healthcare organizations representing 14 hospitals across 5 provinces participated in the development of the scorecard. Two organizations that participated in the inaugural conference were not able to sustain their participation and one organization joined the project subsequent to the first conference.

Quality indicators were selected using nominal group technique, a structured method for generating and narrowing down a list of choices [10]. Following the conference, the group maintained discussions on a regular basis through a combination of email, teleconference calls, webinars, and internet groups. Candidate quality indicators were proposed, developed, and refined using the principles developed at the original conference. The feasibility of the project was demonstrated with initial data collection and reporting using one indicator from each of the selected domains.

A second conference was held on June 6-7, 2007, to review and finalize the list of quality indicators. The process was led by a certified facilitator using established techniques for consensus building and prioritization that occurred over several rounds [10]. The first round identified any undisputed quality indicators. There were five candidate indicators that all participants agreed met all the selection criteria. The second round used cumulative voting. Participants received a total of 5 votes that could be applied in any number to any number of candidate indicators. The top four candidate indicators from this round were selected. In the third round, each participant assigned a weight to each criterion to the remaining candidate indictors. The resulting prioritization matrix led to the selection of nine more indicators. Two more indicators (patient/family satisfaction and nurse absenteeism) were added based on the defined domains, bringing the total to 20 quality indicators. Some potential indicators that were considered but not ultimately chosen included delay in emergency admission, cancelled surgery, elevation of the head of the bed, deep venous thrombosis prophylaxis, use of sedation and restraints, appropriateness of blood transfusions, nutrition, treatment of sepsis, nutrition, use of a pain scale, procedure related complications, ICU acquired* C. difficile* infection, decubitus ulcers, length of stay of ICU decedents, admissions from other hospitals, and repatriation rate. Postextubation respiratory failure was added unanimously during a June 2007 team conference call based on previous discussions. Ventilated patient flow was added to complement patient flow in October 2008 bringing the total to 22 quality indicators (Table 1).

Detailed operational definitions were developed for each quality indicator that included the domain represented, how it is reported, the reporting period, significance of the indicator, derivation, details of data collection, considerations and assumptions in its measurement or derivation, data display, benchmark or goal, revision notes, and references. Abbreviated definitions are shown for all quality indicators in Appendix A and an example of a detailed operational definition is shown in Appendix B.

### 2.2. Data Display

In September 2007, a central, custom web-based application written specifically for this project was implemented to demonstrate how central data upload and data display could work. Participating centres uploaded their aggregate data files on the password protected, limited access website. Individual patients were not identifiable. All work was carried out in accordance with the requirements of the Research Ethics Review Board of each participating site. Local data management centres were responsible for ensuring that the submitted data was clean and consistent with the current definitions of the project.

**Figure 1:**
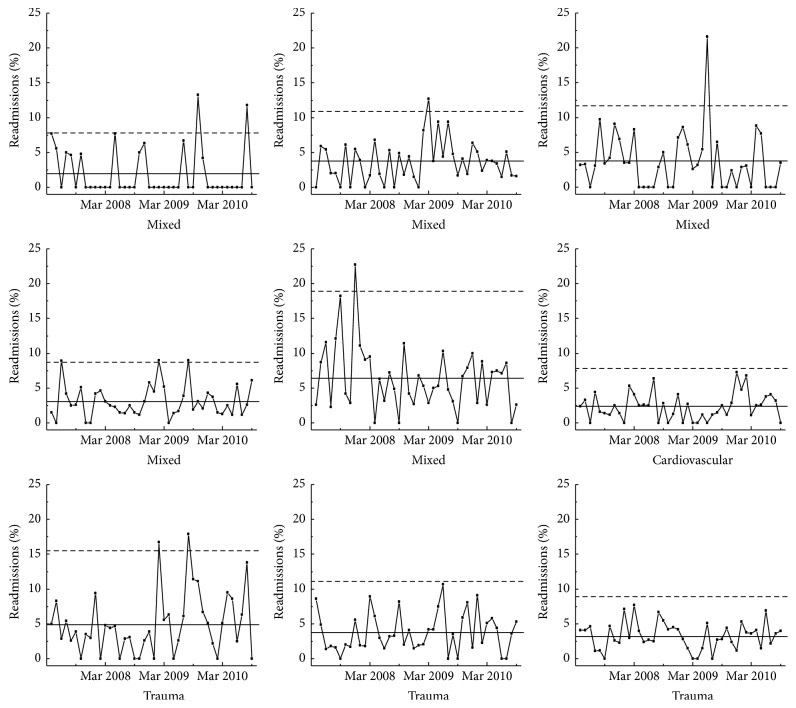
Percent readmissions per month as p-charts in small multiples. Statistical process control p-chart of readmission rate over time shown for 9 units as small multiples. The mean is shown as a solid line and the upper control limit (3*σ*) as the dashed line.

**Figure 2:**
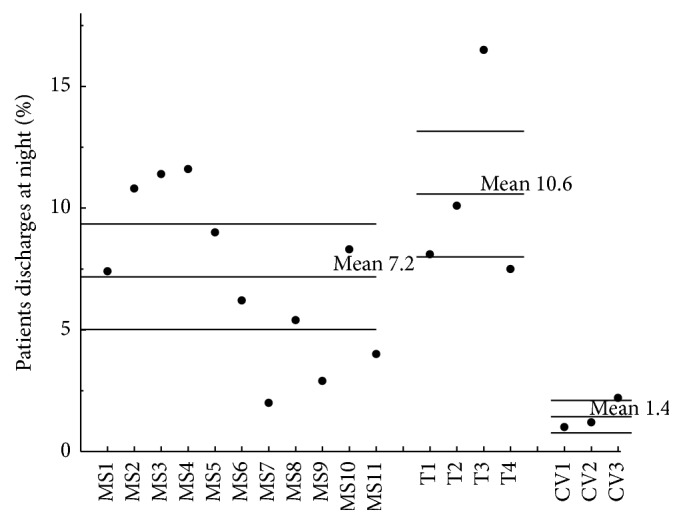
Percent of patients discharged at night. x-bar statistical process control chart with rational subgrouping by unit type (from left to right: mixed medical surgical (MS), trauma (T), and cardiovascular (CV)). The upper and lower control limits are set at 3*σ*.

Statistical process control (SPC) charts were used as the primary mode of data sharing and display. SPC charts have been used extensively by industry for quality control and are becoming increasingly common in medical quality improvement applications [11, 12]. Data from a baseline period are used to identify the usual variation inherent in the current system. Various statistical rules are then applied to time-series or average data to detect when results are significantly different from the usual variation. When applied in real or near-real time, this method avoids wasting time and resources to examine variation that is expected within that system but also identifies data points that should be investigated. SPC charts can also be used to compare average performance between units. An example of the type of SPC charts used is shown in Figures 1 and 2.

Centres could view their quality indicators as well as those of other participating sites. Individual ICUs were identified on the website by a code known to all participants. The types of control charts available for data display included p-charts for individual units over time and x-bar charts for comparisons between units. The ongoing review and discussion of comparative data led to further refinements in the operational definitions and data display. Additional ICU descriptors were added around case mix and teaching status to help units choose potential peers. Different systems for severity of illness adjustment were in use by the participating organizations. A workshop in February 2008 reviewed several validated measures of severity of illness (APACHE II and IV, SAPS, ICNARC, MODS, SOFA, and MPM) without reaching agreement. The choice of a common severity of illness measure was deferred. A meeting in November 2008 was used to further review the operational definitions, data submission, and data display.

### 2.3. User Satisfaction

In December 2008, participants made presentations of representative data from the project to their respective ICU medical directors, nursing leadership, and hospital administrators. This was immediately followed by a request to the leadership to complete an end-user satisfaction survey. Using factor analysis, Doll and Torkzadech [13] identified and validated five components of end-user computing satisfaction: content, accuracy, format, ease of use, and timeliness. We administered four questions, focusing on the components of content and format. Forty leaders (82%) completed the survey. Thirty-one of the respondents (78%) answered that the new reporting system was better than their previous one, with the main reason being that it was easier to access and see the data (74% of respondents). The most common question raised by the new reporting system was how to interpret the data (45% of respondents). There were 20 responses as to how the system could be improved: offer training in interpretation of the data (40%), ensure data quality and consistent definitions (25%), and add more measures (20%) and more benchmarking (15%).

The results of the survey supported the decision to use control charts for data display but also pointed out that education would be needed for end-users to get maximum value from them. Results also confirmed and reinforced that there was a desire for measures and methodology that would allow for comparisons with others.

### 2.4. Participating Units and Data Collection

Data submission, review, and revision continued with an ongoing iterative process. The last major revision to the operational definitions was at a conference in June 2011.

Participating ICUs were “closed” with all patients cared for by a consultant intensivist with 24-hour availability and daily multidisciplinary rounds. All critical care admissions were captured in local data sets that contain patient demographic information, admission diagnoses, a severity of illness measure, admission and discharge times, ventilation days, and mortality. Data on nosocomial infections was obtained from the local infection control service and data on nursing work hours from each institution's financial office. Collection of data on indicators such as extubation failure, patient satisfaction, and organ donation was obtained through processes that varied from one site to another. As much as possible, the submission of component variables was favored over the submission of a rate. For example, rather than sites submitting the rate of unplanned extubations, they instead submitted the number of unplanned extubations and the sum of invasive mechanical ventilation and the rate was calculated centrally. This allowed more flexibility in deriving other indicators and in checking for internal consistency.

For purposes of this paper, all sites resubmitted data consistent with the latest operational definitions to a central office as either Excel spreadsheets (Microsoft Corporation, Redmond, WA) or SAS data files (SAS Institute, Cary, NC) which were merged into a primary dataset. Data was checked for completeness, internal consistency, and suspected outlying data by one of the authors. Comparative data was circulated and participating sites were required to validate or correct their data. New variables were derived from submitted data as defined in the operational definitions. For example, wasted bed days were derived by dividing the number of avoidable days by the number of days in the month.

Intensive care units were divided into three groups based on their case mix. Cardiovascular ICUs admitted postcardiovascular surgery patients exclusively. Trauma ICUs admitted trauma and neurosurgical patients in addition to general medical and/or surgical patients. Mixed ICUs admitted a mix of general medical, surgical, and cardiac patients. One mixed ICU also admitted postoperative cardiovascular surgery patients.

### 2.5. Analysis

Continuous variables were expressed as the mean and standard deviation, with the exception of the number of beds which was expressed as the median and range. The ICU length of stay was expressed as median and interquartile range in addition to mean. Statistical analysis was performed using SAS 9.3 (SAS Institute, Cary, NC). Graphs were created using OriginPro 7 (OriginLab Corporation, Northampton, MA).

## 3. Results

Eighteen intensive care units (11 mixed, 4 trauma, and 3 cardiovascular), of which 78% were teaching units, submitted data on admissions from April 1, 2007, to September 30, 2010, inclusive. Less than half of the units were able to submit data on patient and family satisfaction and staff turnover, overtime, and absenteeism, and results for these indicators will not be described. Data for monthly variables was complete with the exception of 3 indicators. Avoidable days were missing from one cardiovascular unit for the entire study period; therefore wasted bed days could not be calculated. Ventilator utilization ratio also uses a correction for avoidable days. However, as the uncorrected ventilator utilization ratio was already 0.98 for this unit, the uncorrected ratio was used. Mechanical ventilation days were partially missing from 3 units (23 months, 3% of the data) and occupancy data from one unit (2 months, 0.3% of the data) was missing and was treated as missing.

Not all units collected data on the selected quarterly indicators prior to 2009. This does not represent a gap in data collection, but a lag in the implementation of newly agreed upon indicators. The average therefore is expressed as the mean for the last 4 quarters of the study period (fourth calendar quarter of 2009 to third calendar quarter of 2010). There were 6 instances (1.3% of quarterly data points) where data was missing from the third quarter of 2010. Data from the third calendar quarter of 2009 was used instead so as to be representative of 12 continuous months.

The median size of participating ICUs was 12 beds, with a large range of unit sizes (6–27 beds). Over the 3.5-year study period, there were 49,762 admissions with 275,173 patient days.  Figure 1 is an example of a chart that shows the percent of readmissions per month over the entire study period as p-charts in small multiples for nine of the participating units. Small multiples are a way to display different slices of a data set. They can be helpful in revealing patterns and making comparisons.  Figure 2 is an example of an x-bar statistical process control chart with rational subgrouping based on the type of the unit showing marked variation between units in the rate of night discharges. Similar process control charts, using either x-bar or p-charts, were used to report all indicators on a regular basis. The average performance on the developed ICU quality indicators is shown in Table 2.

**Figure 3:**
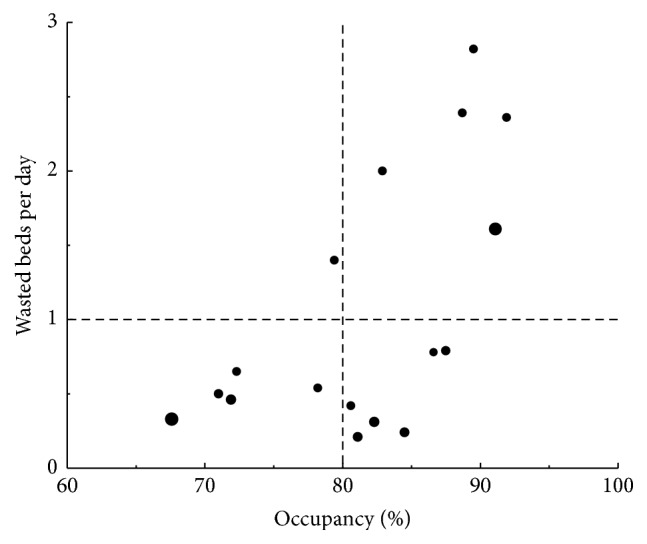
Intensive care unit occupancy versus wasted ICU beds per day. Average of the monthly occupancy is plotted against the average number of wasted ICU beds per day. Bubble size is proportional of patient flow (patients/bed/year). Vertical dashed line is at 80% occupancy and horizontal dashed line at 1 wasted bed per day.

The units that participated in this project care for sick patients as evidenced by a ventilator utilization ratio of 0.72 ± 0.16 and hospital mortality of 20.1 ± 8.2%. They are also very active with patient flow ranging from 46.6 ± 1.2 patients/bed/year in trauma units to 98.7 ± 17.0 patients/bed/year in cardiovascular units. Occupancy is a frequently used indicator of unit activity. The participating units had an average occupancy of 81 ± 7%. However avoidable days represented 8.8 ± 3.6% of patient days amounting to 1.09 ± 0.85 wasted ICU beds per day.  Figure 3 is an example of a composite measure showing the number of wasted ICU beds versus average monthly occupancy by unit. A vertical dashed line is drawn at 80% occupancy, a common target for ICUs. The horizontal dashed line is drawn at one wasted bed per day, a level that was felt to represent a significant waste of human resources. Units in the upper right quadrant have high occupancy, with a number of beds occupied by patients no long requiring ICU care. Units in the lower left quadrant, however, maintain a low level of wasted beds, even at low occupancy.

## 4. Discussion

We have developed and demonstrated the successful implementation of a process for leveraging existing data and knowledge into a system for monitoring and comparing intensive care unit performance. We achieved a high level of compliance with data submission and satisfaction reported by end-users.

The Quality Improvement in Critical Care Project team was able to agree upon 22 quality indicators spanning 6 quality of care domains. We attribute our success with this selection process to being a relatively homogeneous group (academic institutions in a public funded health care system with physician specialty training under a single national specialty organization), the agreement on principles for quality indicator selection, and a pilot phase that demonstrated the initial feasibility of our approach. In addition, our participants came from centres with existing databases so the ability to collect and submit the data under consideration was known. Multiple cycles of discussion coupled with the review of real data were required to uncover all of the potential variation in the data definitions and implementation or data management processes between ICUs. The importance of detailed, iterative review to address this variation cannot be overstated or overemphasized when it comes to comparing data between different institutions and jurisdictions. For example, even something as fundamental as what constitutes an ICU bed required significant discussion. Explicit operational definitions are essential.

Our domains are similar to the aims for improvement from the Institute of Medicine [14] with the exception of equity and the addition of staff work life. We recognize that socioeconomic status, geographic distance from an urban centre, and gender are important possible sources of inequity even in a publicly funded health care system such as Canada's. Inclusion of equity as a domain was beyond the scope of this initial project. We did include indicators for staff work life as a domain since intensive care is an environment with a high risk of burnout that can negatively impact the quality of care [15].

The European Society of Intensive Care Medicine recently agreed upon 9 quality indicators of which approximately half are related to ICU organization and structure [9]. Three of the European Society's indicators (readmission, rate of central venous catheter-related bloodstream infections, and rate of unplanned endotracheal extubations) are also included our quality indicators.

Strengths of this study include the participation of multiple institutions and regions from across the country; participation of knowledge users, clinicians, and improvement experts; and the demonstration that existing data sources can be effectively combined in statistical control charts that users judged to be superior to previously available reports.

One limitation was the lack of severity of illness adjustment. Adjusting for severity of illness requires additional resources for data collection and adds complexity to the derivation of the indicators. How important adjusting for severity of illness is in the interpretation and use of indicators such as mortality, patient flow, length of stay, and readmission for purposes of quality improvement is unclear and the subject of future work. The current lack of severity adjustment is less of a limitation when monitoring these quality indicators over time since illness severity will be relatively stable within individual ICUs. Quality Indicators such as ventilator utilization ratio and ICU mortality can be used to help guide the selection of appropriate peer groups for interunit comparisons. Also, severity of illness is only one of many factors that might need to be considered when exploring any signal produced by the data.

We also identified challenges with the collection of indicators relating to patient satisfaction and staff work life that require establishing entirely new processes of data collection directly from patients/families and from hospital finance, respectively. Although we developed and implemented a web-based data submission and reporting system, this was on a demonstration platform and did not have integrated data checking or validation tools. Thus, we relied on a manual process for this and the current report is based on the final data submission, rather than real-time data that would be useful for the purposes of performance or quality improvement.

In this paper we have reported the average performance of our indicators. As the participants in the project represent large urban centres with highly motivated individuals, it is unclear to what extent the results are representative of the rest of Canada. We have explored the use of statistical process control charts and have found them to be a useful and acceptable way to display the data to our intended audience.

The Quality Improvement in Critical Care Project has succeeded with 5 Canadian health care organizations to develop and implement a system for sharing and displaying a common set of performance data. The process used a set of predetermined principles and series of consensus methods to develop 22 detailed operational definitions for quality indicators representing 6 domains of intensive care unit function. We believe that the use of these quality indicators and statistical process control charts will be a powerful tool for benchmarking and using measurement to lead to improvement in patient safety and clinical processes. The methods that we have described can be applied by other healthcare groups.

## Figures and Tables

**Table 1:** Quality indicators and their domains.

*Safe *	
Unplanned extubation^*∗*^	
Readmission to intensive care unit	
Incidence of ventilator associated pneumonia^*∗*^	
Incidence of central line-related bloodstream infections^*∗*^	
Incidence of intensive care unit-acquired methicillin-resistant *Staphylococcus aureus* ^*∗*^	
Prevalence of intensive care unit-acquired methicillin-resistant *Staphylococcus aureus* ^*∗*^	
*Timely *	
Occupancy	
Intensive care unit discharges that occur at night	
*Efficient *	
Avoidable days in intensive care unit	
Patient flow	
Ventilated patient flow	
Ventilator utilization ratio	
Interfacility patient transfers	
*Effective *	
Intensive care unit length of stay	
Extubation failure rate^*∗*^	
Intensive care unit mortality	
Hospital mortality	
Consent rate for solid organ donation^*∗*^	
*Patient/family satisfaction *	
Patient/family satisfaction	
*Staff work life *	
Staff turnover	
Overtime	
Absenteeism	

^*∗*^Indicators calculated per calendar quarter.

**Table 2:** Unit characteristics and average performance.

Measure		All	Mixed	Trauma^a^	CV
Units	*n*	18	11	4	3
Beds	med (range)	12 (6–27)	10 (6–24)	25 (11–27)	12 (10–14)
Annual admissions	mean (sd)	790 (354)	599 (250)	1028 (342)	1173 (202)
Annual patient days	mean (sd)	4368 (2395)	3605 (1896)	7119 (2655)	3495 (848)

Monthly indicators-42 months					
Readmissions (%)	mean (sd)	3.3 (1.4)	3.5 (1.5)	4.1 (0.7)	1.8 (0.5)
Occupancy (%)	mean (sd)	81 (7)	80 (6)	86 (5)	78 (12)
Nighttime discharges (%)	mean (sd)	7.0 (4.3)	7.2 (3.4)	10.6 (4.1)	1.4 (0.6)
Avoidable days (%)	mean (sd)	8.3 (3.6)^b^	7.8 (3.6)	9.1 (3.1)	9.1 (6.4)^b^
Avoidable days (beds/d)	mean (sd)	1.05 (0.87)^b^	0.75 (0.64)	1.90 (1.04)	1.0 (0.9)^b^
Ventilator utilization	mean (sd)	0.72 (0.16)	0.71 (0.11)	0.83 (0.07)	0.61 (0.31)
Ventilated patient flow (pt/bed/y)	mean (sd)	42.0 (18.5)	33.3 (7.2)	39.3 (2.7)	77.3 (18.4)
Patient flow (pt/bed/y)	mean (sd)	58.3 (20.3)	51.6 (7.0)	46.6 (1.2)	98.7 (17.0)
Interfacility transfers (%)	mean (sd)	5.1 (3.8)	6.8 (3.5)	3.7 (2.6)	0.4 (0.3)
Length of stay (days)	mean (sd)	5.6 (1.6)	5.9 (1.1)	6.8 (0.6)	3.0 (0.6)
Length of stay (days)	med (IQR)	3.0 (1.0)	3.1 (0.9)	4.0 (0.7)	1.3 (0.3)
ICU mortality (%)	mean (sd)	14.3 (6.8)	16.2 (4.9)	17.5 (4.9)	2.9 (1.6)
Hospital mortality (%)	mean (sd)	20.1 (8.2)	23.2 (4.5)	23.5 (3.4)	4.3 (2.5)

Quarterly indicators-one year 2009Q4-2010Q3					
Unplanned extubation/1000 ventilator days	mean (sd)	4.8 (3.5)^c^	5.3 (3.9)	4.9 (1.9)	0.8 (0)^c^
Incidence VAP/1000 ventilator days	mean (sd)	4.2 (2.0)^c^	3.9 (2.0)^b^	4.8 (2.4)^b^	4.8 (2.5)
Incidence CLBSI/1000 line days	mean (sd)	1.1 (0.9)^b^	1.0 (0.8)	1.2 (0.9)	1.6 (1.6)^b^
MRSA on admission/1000 patients	mean (sd)	25.9 (15.5)^b^	31.3 (13.2)^b^	29.4 (11.6)	3.4 (3.1)
ICU acquired MRSA/1000 patient days	mean (sd)	0.90 (0.84)	0.77 (1.02)^b^	1.26 (0.31)	0.82 (0.74)
Extubation failure rate (%)	mean (sd)	2.7 (1.5)	2.8 (1.3)	3.5 (1.6)	1.0 (0.9)
Organ donation	donor/potential	58/102	28/62	30/39	0/1

^a^These units admit medical and/or surgical patients in addition to trauma patients.

^b^Data missing from one unit.

^c^Data missing from two units.

## Appendix

### A. Abbreviated Operational Definitions of Quality Indicators

*Unplanned Extubation*

Number of unplanned extubations per 1000 invasive mechanical ventilation days. (See also Appendix B.)

*Readmission to ICU*

Number of patients with an unplanned readmission to ICU within 72 hours of ICU discharge within the same hospitalization, calculated as a percent of live discharges.

*Incidence of Ventilator Associated Pneumonia (VAP)*

Number of pneumonias occurring in patients requiring a device to assist respiration through a tracheostomy or endotracheal tube. The device must have been in place within the 48-hour period before the onset of infection and for at least 2 consecutive days, reported as VAP per 1000 ventilator-days.

*Incidence of Central Line-Related Bloodstream Infections (CLBSI)*

The number of cases with a laboratory confirmed bloodstream infection associated with a central venous catheter expressed per 1000 line days.

*Prevalence of Methicillin-Resistant S. aureus (MRSA)*

Number of patients identified as MRSA positive from surveillance or clinical samples obtained within 24 hours of ICU admission, calculated as cases per 1000 ICU discharges.

*Incidence of ICU Acquired Methicillin-Resistant S. aureus (MRSA)*

Number of patients who were MRSA negative on admission with subsequent isolation of MRSA from any sample obtained 24 hours or more after ICU admission, calculated as cases per 1000 ICU discharges.

*Occupancy*

Average occupancy is calculated as the sum of the average maximum census and average minimum census divided by twice the number of ICU beds. An ICU bed is defined as the number of beds regularly available for patient care, regardless of staffing. Occupancy is expressed as percent.

*ICU Discharges That Occur at Night*

Number of patients discharged alive to a ward, step-down, high-dependency, high observation, or another non-ICU patient area in the same hospital, between the hours of 22:00 and 06:59, calculated as a percent of all live ICU discharges.

*Avoidable Days in ICU*

The amount of time that a patient occupies an ICU bed when ICU care is no longer required. The amount of time that patients occupy an ICU bed for more than 4 hours after a transfer order is written is considered avoidable. Avoidable days (24 hours) are expressed as a percent of total patient days.

*Patient Flow*

Patient flow indicates patient throughput and is a reflection of case mix and efficiency. It is calculated as the number of admissions per bed per year.

*Ventilated Patient Flow*

Mechanical ventilation is the primary characteristic that distinguishes a patient requiring full critical care services from one requiring only high dependency or an intermediate level of care. It is expressed as the number of patients receiving mechanical ventilation (invasive or noninvasive for an acute indication) per ICU bed per year.

*Ventilator Utilization Ratio*

Ratio of ventilator days (invasive or noninvasive for an acute indication) to total patient days corrected for avoidable days.

*Interfacility Patient Transfers*

Refers to transfers to another hospital that occur during an ICU admission. Transfers may be required for medical reasons (need for a medical service/intervention not available at the initial hospital) or as part of ICU bed management, calculated at percent of live ICU discharges.

*ICU Length of Stay*

Calculated from the date/time of ICU admission and discharge. The length of stay encompasses avoidable days.

*Extubation Failure Rate*

The number of patients requiring reintubation within 48 hours of a planned extubation, calculated as percent of invasively ventilated patients.

*ICU Mortality*

Number of patients who died while under the care of the ICU team, calculated as percent of all ICU discharges.

*Hospital Mortality*

Number of patients that died while under the care of the ICU team or following discharge from ICU during the same hospitalization, calculated as percent of all ICU discharges.

*Consent Rate for Solid Organ Donation*

The number of neurologic determination of death (NDD) patients for whom consent was obtained for solid organ donation. Calculated as a percent of eligible neurologic determination of death (NDD) patients.

*Patient/Family Satisfaction*

Total score and decision-making and care subscales from the Family Satisfaction-24 survey.

*Staff Turnover*

Number of nurses leaving ICU, calculated as percent of total number of nurses working in the ICU.

*Overtime*

Number of nursing overtime hours, calculated as a percent of total hours worked.

*Absenteeism*

Number of nurses sick hours, calculated as percent of total number of hours.

### B. Example of a Detailed Operational Definition of a Quality Indicator

*Quality Indicator*

Unplanned extubation.

*Domain*

Safe.

*Reported As*

Number of unplanned extubations per 1000 invasive mechanical ventilation days.

*Reporting Period*

Quarterly.

*Definition*

Unplanned extubation is the unscheduled removal of an artificial airway (endotracheal or tracheostomy tube) due to accidental dislodgement or patient self-extubation. The patient need not be ventilated at the exact time of the event (e.g., on t-piece or tracheal mask).

*Significance*

Unplanned extubation may result in patient harm and prolonged length of stay due to loss of the airway and the risks associated with recapture.
Putative factors in unplanned extubation include inadequate/inappropriate:
  - positioning, length, or fastening of artificial airways,
  - management of analgesia, sedation, and delirium,
  - vigilance, nurse : patient ratio, and the use of physical restraints.

A significant and/or sustained increase in unplanned extubations should lead to the review of these factors.

*Derivation*

*Numerator*

Number of unplanned extubations in the reporting period.

*Denominator*

Sum of invasive mechanical ventilation days in the reporting period:
  (B.1) Calculation=number  of  unplanned  extubationssum  of  invasive  mechanical  ventilation  days×1000.

*Data Collection*

Unplanned extubations may be captured from nursing or respiratory therapist flow sheets or from review of patient incident/hazard/safety reports. Each institution is responsible for maintaining a process for recognizing and documenting all unplanned extubations. Invasive ventilation days may be captured by counting the number of mechanically ventilated patients in the ICU at approximately the same time every day and assigning one day of mechanical ventilation to each of these patients (similar to the VAP measure).

*Considerations and Assumptions*

The number of days when a patient has an artificial airway but is not on invasive mechanical ventilation is not readily available and is not included. While this will lead to an overestimate of the rate of unplanned extubation, the magnitude is expected to be small.

*Data Display*

XmR statistical process control run chart with 3 sigma limits.

*Benchmark/Goal*

Best reported rates in the literature are <5%.

*Revision Notes*

Current version was in May 2012.
Previous versions were in June 2011.
Key changes are none and organizational and text edits.

*Additional Resources*

Bouza, E. Garcia, M. Diaz, E. Segovia, and I. Rodriguez, “Unplanned extubation in orally intubated medical patients in the intensive care unit: a prospective cohort study,” Heart & Lung, vol. 36, no. 4, pp. 270–276, 2007.
R. S. Ream, K. Mackey, T. Leet et al., “Association of nursing workload and unplanned extubations in a pediatric intensive care unit,” Pediatric Critical CareMedicine, vol. 8, no. 4,pp. 366–371, 2007.
Krayem, R. Butler, and C.Martin, “Unplanned extubation in the ICU: impact on outcome and nursing workload,” Annals of Thoracic Medicine, vol. 1, no. 2, pp. 71–75, 2006.
P. Moons, K. Sels, W. De Becker, S. De Geest, and P. Ferdinande, “Development of a risk assessment tool for deliberate selfextubation in intensive care patients,” Intensive Care Medicine, vol. 30, no. 7, pp. 1348–1355, 2004.
S. K. Epstein, M. L. Nevins, and J. Chung, “Effect of unplanned extubation on outcome of mechanical ventilation,” American Journal of Respiratory and Critical Care Medicine, vol. 161, no. 6, pp. 1912–1916, 2000.
T. Boulain, G. Bouachour, J. P. Gouello et al., “Unplanned extubations in the adult intensive care unit: a prospective multicenter study,” American Journal of Respiratory and Critical Care Medicine, vol. 157, no. 4, pp. 1131–1137, 1998.
P. M. Atkins, L. C. Mion, W. Mendelson, R. M. Palmer, J. Slomka, and T. Franko, “Characteristics and outcomes of patients who self-extubate from ventilatory support: a case-control study,” Chest, vol. 112, no. 5, pp. 1317–1323, 1997.

## Additional Points

Quality improvement in critical care project contributors are the following: Department of Critical Care Medicine, University of Calgary, Calgary, AB: Paul J. E. Boiteau, M.D.; Carla A. Chrusch, M.D., M.S.; Reza Shahpori, M.S; Improvement Associates, Edmonton, AB: Bruce Harries, MBA; Department of Medicine, University of Western Ontario, London, ON: Claudio M. Martin, M.D., M.S.; London Health Sciences Centre, London, ON: Fran Priestap, M.S.; Department of Medicine, McGill University, Montreal, QC: Denny Laporta, M.D.; Vancouver Coastal Health, Vancouver, BC: Shelly Fleck, EMBA; Department of Medicine, University of Manitoba, Winnipeg, MB: Kendiss Olafson, M.D., MPH; Julie Mojica, M.S. Additional contributors are the following: London Health Sciences Centre, London, ON: Judy Kojlak, MScN (*interpretation of data and obtaining funding*); Providence Health Care, Vancouver, BC: Bonita Elliott, BScN (*interpretation of data*); Departments of Nursing and Adult Critical Care, McGill University and Jewish General Hospital, Montreal, QC: Barbara Verhoef, BScN (*acquisition of data*). Participating hospitals are the following: Calgary, AB: Rockyview General Hospital, Peter Lougheed Centre, and Foothills Medical Centre (2 ICUs); London, ON: Victoria Hospital and University Hospital (2 ICUs); Montreal, QC: Jewish General Hospital; Vancouver, BC: Vancouver General Hospital and St. Paul's Hospital; Winnipeg, MB: Concordia Hospital, Grace Hospital, Seven Oaks General Hospital, Victoria General Hospital, Health Sciences Centre (2 ICUs), and St. Boniface Hospital (2 ICUs).

## References

[1] Ewart G. W., Marcus L., Gaba M. M., Bradner R. H., Medina J. L., Chandler E. B. (2004). The critical care medicine crisis: a call for federal action. *Chest*.

[2] Levy M. M., Dellinger R. P., Townsend S. R. (2010). The Surviving Sepsis Campaign: results of an international guideline-based performance improvement program targeting severe sepsis. *Critical Care Medicine*.

[3] Institute of MedicineInstitute of Medicine Report (1999). *To Err is Human: Building a Safer Health System*.

[4] Barnato A. E., Kahn J. M., Rubenfeld G. D. (2007). Prioritizing the organization and management of intensive care services in the United States: the PrOMIS Conference. *Critical Care Medicine*.

[5] Pronovost P. J., Sexton J. B., Pham J. C., Goeschel C., Winters B. D., Miller M. R. (2009). Measurement of quality and assurance of safety in the critically Ill. *Clinics in Chest Medicine*.

[6] Slonim A. D., Pollack M. M. (2005). Integrating the Institute of Medicine's six quality aims into pediatric critical care: relevance and applications. *Pediatric Critical Care Medicine*.

[7] Scanlon M. C., Mistry K. P., Jeffries H. E. (2007). Determining pediatric intensive care unit quality indicators for measuring pediatric intensive care unit safety. *Pediatric Critical Care Medicine*.

[8] Pronovost P. J., Berenholtz S. M., Ngo K. (2003). Developing and pilot testing quality indicators in the intensive care unit. *Journal of Critical Care*.

[9] Rhodes A., Moreno R. P., Azoulay E. (2012). Prospectively defined indicators to improve the safety and quality of care for critically ill patients: a report from the task force of Safety and Quality of the European Society of Intensive Care Medicine (ESICM). *Intensive Care Medicine*.

[10] Scholtes P., Joiner B., Streibel B. (2003). *The Team Handbook*.

[11] Nicolay C. R., Purkayastha S., Greenhalgh A. (2012). Systematic review of the application of quality improvement methodologies from the manufacturing industry to surgical healthcare. *British Journal of Surgery*.

[12] Thor J., Lundberg J., Ask J. (2007). Application of statistical process control in healthcare improvement: systematic review. *Quality & Safety in Health Care*.

[13] Doll W. J., Torkzadech G. (1988). The measurement of end-user computing satisfaction. *MIS Quarterly*.

[14] Institute of Medicine Committee on Quality of Health Care in America (2001). *Crossing the Quality Chasm: A New Health System for the 21st Century*.

[15] Embrico N., Papazian L., Kentish-Barnes N., Pochard F., Azoulay E. (2007). Burnout syndrome among critical care healthcare workers. *Current Opinion in Critical Care*.

